# A Framework to Avoid Significance Fallacy

**DOI:** 10.7759/cureus.40242

**Published:** 2023-06-11

**Authors:** Alessandro Rovetta

**Affiliations:** 1 Research and Disclosure Division, R&C Research, Bovezzo (BS), ITA; 2 Technological and Scientific Research, Redeev Srl, Naples, ITA

**Keywords:** causality, decision-making, effect size, hypothesis testing, p-value, reproducibility, research methods, significance fallacy, statistical significance, study design

## Abstract

This manuscript presents a concise approach to tackle the widespread misuse of statistical significance in scientific research, focusing on public health. It offers practical guidance for conducting accurate statistical evaluations and promoting easily understandable results based on actual evidence. When conducting a statistical study to inform decision-making, it is recommended to follow a step-by-step sequence while considering various factors. Firstly, multiple target hypotheses should be adopted to assess the compatibility of experimental data with different models. Reporting all P-values in full, rounded in order to have a single non-zero significant digit, enhances transparency and reduces the likelihood of exaggerating the state of the evidence. Detailed documentation of the procedures used to evaluate the compatibility between test assumptions and data should be provided for rigorous assessment. A descriptive evaluation of results can be aided by using statistical compatibility ranges, which help avoid misrepresenting the evidence. Separately evaluating and reporting statistical compatibility and effect size prevents the magnitude fallacy. Additionally, reporting measures of statistical effect size enables evaluation of sectoral relevance, such as clinical significance. Multiple compatibility intervals, such as 99%, 95%, and 90% confidence intervals, should be reported to allow readers to assess the variation of P-values based on the width of the interval. These recommendations aim to enhance the robustness and interpretability of statistical analyses and promote transparent reporting of findings. The author encourages journal adoption of similar frameworks to enhance scientific rigor, particularly in the field of medical science.

## Introduction

The abuse and misuse of statistical significance and hypothesis testing in scientific research are well-known and widely debated concepts [1]. In sensitive areas such as public health, errors of this kind can have serious consequences, such as the use of ineffective drugs. Significance misconceptions can lead authors to exaggerate the degree of evidence found. Despite its importance, this issue is often overlooked, ignored, or even rejected by a large part of the scientific community. Such behavior may be due to a lack of easy interpretation of statistical significance, inadequate university teaching, cognitive distortions, and harmful practices such as publication bias and the pressure to publish or perish [1-3]. In such a context, the current manuscript aims to provide a simple and practical summary of the procedures to be adopted to carry out correct statistical evaluations, thus presenting results that are easily interpretable and weighted on the real degree of evidence found. The goal is not to further enrich an already saturated discussion, but to guide the reader based on what is known and consolidated in the literature, although heavily underestimated. The author hopes that this or similar frameworks will be required by peer-reviewed journals for the benefit of science, especially medical science.

## Technical report

First premise: methodological validity of the study

Considerations on statistical evidence are relevant if and only if all initial procedures (e.g., study design, data collection, experiment conduct, etc.) have been carried out correctly, that is, ensuring sufficient levels of neutrality, competence, attention, and collaboration [1]. Since it is not easy to determine or disclose evidence on methodological validity, it is necessary to consider that no study based on a single investigation can provide conclusive evidence on a phenomenon and/or lead to sufficiently informed practical decisions. However, even in the single case, there is a substantial informative difference between research that favors transparency and reproducibility and those that do not.

Second premise: statistical effect size

The concept of statistical significance is directly linked to the degree of surprise (how unexpected a result is) compared to the prediction of a statistical model, but it does not provide clear and unambiguous information on the magnitude of the statistical effect because this relationship is confused, at best, by the size of the dataset [2]. Therefore, in general, these two aspects are separate.

Fisher's approach (single-study conclusions)

When conducting a single study, the concept of statistical significance can be evaluated using Fisher’s approach [1,4]. Given a certain target hypothesis (e.g., there is no difference between the average cholesterol values before and after treatment), a test is adopted to evaluate the “statistical relevance” of the experimental data we have observed in relation to this hypothesis. Assuming that the chosen test is the most suitable for the purpose, the measure of this relevance is called the P-value (P). Before formally defining the P-value, it is necessary to reiterate a couple of essential facts. The first fact (F1) is that the P-value is calculated assuming that all hypotheses of the model, including the target hypothesis, are true. The second fact (F2) is that the P-value gives clear information on the relationship between the target hypothesis and the experimental data only when all other test assumptions (e.g., distributive normality in parametric tests) are sufficiently satisfied.

Considering F1 and assuming F2, the P-value can be defined as a continuous measure of the compatibility between the target hypothesis we have chosen and the experimental data we have observed. The P-value ranges from a minimum value of 0 (very low compatibility) to a maximum value of 1 (very high compatibility) [1].

Neyman-Pearson approach (conclusions from multiple studies)

When analyzing the results of multiple valid studies (see previous sections), the Neyman-Pearson approach, also known as hypothesis testing, allows for limiting the proportion of type I errors (false positives) and type II errors (false negatives) [4]. This is done by establishing two thresholds, α and β, both between 0 and 1. Some fundamental facts must be emphasized. In addition to the target hypothesis, an alternative hypothesis must be established (F3). The error thresholds α and β must be set a priori (before conducting all studies) because they determine the sample size (F4). In order for α and β to be informative, it is necessary to ensure that all experiments are repeated under conditions capable of maintaining their validity (both methodological and statistical) (F5). This approach provides global information, i.e., only on the entire set of experiments and never on specific ones (F6). For each individual study, the target hypothesis is arbitrarily rejected - in favor of the alternative hypothesis - only when P < α (F7).

Considering F3, F4, F6, and F7, and assuming F5, it is plausible to think of committing, in total, about α% type I errors and about β% type II errors. This means that we can roughly know how much we are wrong in multiple repetitions but not where we are wrong (i.e., we cannot know for which of the individual studies a wrong decision has been made).

Simple operating framework

When conducting a single statistical study with the aim of informing a decision - also based on evidence of other nature (e.g., biological, psychological, etc.) or research in general - it is suggested to adopt the following sequence step by step, keeping in mind all the considerations made above. Adopt multiple target hypotheses (e.g., mean value = 0, mean value > 0, mean value < 0) and observe the variation of P-values as a function of the latter. In this way, the reader can get a picture of the compatibility of experimental data with various (even contrasting) models (S1). Always report all P-values in full, keeping a single significant digit different from 0 (e.g., P = 0.049 becomes P = 0.05, and P = 0.044 becomes P = 0.04). This increases transparency and interpretability and reduces the likelihood of reporting exaggerations of the state of evidence (since the calculation of the P-value is subject to a wide margin of uncertainty) (S2). Report in detail, in the manuscript or in a supplementary file, all the procedures adopted to evaluate the compatibility between the test assumptions and the experimental data. This gives the reader the opportunity to fully evaluate the statistical validity of the investigation (S3). Use statistical compatibility ranges for descriptive evaluation of results (e.g., from “very weak compatibility” to “very high compatibility”). Some possible solutions are proposed in other literature [5,6]. This reduces the likelihood of communicating exaggerations of the state of evidence (S4). Evaluate, comment on, and report statistical compatibility and statistical effect size separately and independently. This avoids falling into the magnitude fallacy (S5). Always report measures of statistical effect size (e.g., Cohen-Hedges’ g, compatibility intervals, best estimates). This gives the reader the means to evaluate the sectoral relevance (e.g., clinical) of the results (S6). Report multiple compatibility intervals (e.g., 99|95|90-%CI). Some possible solutions are proposed in other literature [5,6]. This gives the reader the opportunity to evaluate the variation of the P-value as a function of the width of the compatibility interval (S7).

## Discussion

This paper highlights the importance of adopting a rigorous and transparent approach when conducting statistical analyses in medical and all decision-based research. Especially when dealing with sensitive areas such as public health, even the above-discussed misinterpretations and errors must be weighed on a cost-benefit function specific to the stakeholders.

By adopting multiple target hypotheses, reporting all P-values in full according to the above-described modalities, adopting statistical compatibility ranges, and providing detailed information on the procedures used to evaluate test assumptions, researchers can reduce the likelihood of exaggerating the state of evidence and ensure that research findings are accurately communicated and interpreted.

Furthermore, the clear distinction between the evaluation of statistical compatibility and statistical and clinical effect sizes can provide valuable information on the sectoral relevance of research findings and help scientists avoid falling into the magnitude fallacy.

This framework is designed to provide a brief and clear list of operations to be performed to inform any public health decision regarding the statistical aspect related to testing. For those who wish to delve deeper into these aspects, the following reading is recommended [7].

## Conclusions

In conclusion, this manuscript presents a practical approach to addressing the misuse of statistical significance in scientific research, with a focus on public health. By following the recommended step-by-step sequence and adopting multiple target hypotheses, reporting all P-values in full, using statistical compatibility ranges, and adopting separate measures of statistical and clinical effect size, researchers can enhance the accuracy and interpretability of their statistical analyses. These recommendations aim to reduce overstatements, promote transparent reporting of findings, and encourage the adoption of similar frameworks by journals to enhance scientific rigor, particularly in the medical field and all decision-based sciences.
